# High conjugated linoleic acid enriched ghee (clarified butter) increases the antioxidant and antiatherogenic potency in female Wistar rats

**DOI:** 10.1186/1476-511X-12-121

**Published:** 2013-08-07

**Authors:** Kathirvelan Chinnadurai, Harpreet Kaur Kanwal, Amrish Kumar Tyagi, Catherine Stanton, Paul Ross

**Affiliations:** 1 Department of Animal Nutrition, Veterinary College and Research Institute, Namakkal, TamilNadu, 637 002, India; 2 Dairy Cattle Nutrition (DCN) Division, National Dairy Research Institute, Karnal, Haryana, 132001, India; 3 Teagasc, Moorepark Food Research Centre, Fermoy, County Cork, Ireland

**Keywords:** High conjugated linoleic acid enriched ghee, Antiatherogenic, Antioxidant, Catalase, Superoxide dismutase, Cholesterol

## Abstract

**Background:**

Hypercholesterolemia and oxidative stress are the main stimulating factors responsible for coronary artery disease and progression of atherosclerosis. Dairy food products are rich in conjugated linoleic acid (CLA) which is considered as an important component due to its potential health benefits such as anticarcinogenic, antiatherogenic, antidiabetic and antiadipogenic properties. In the present study, the effect of CLA enriched ghee on the antioxidant enzyme system and antiatherogenic properties in Wistar rats has been studied.

**Methods:**

Female Wistar rats of 21 days were taken for the study and fed with soybean diet (Control diet), low CLA diet and high CLA ghee diet (treatments) for thirty five days for studying antioxidative enzymes and sixteen weeks in case of antiatherogenic studies.

**Results:**

Feeding of high CLA enhanced ghee during pubescent period in rats lead to an increase in catalase (CAT) and superoxide dismutase (SOD) enzyme activities in blood and increased CAT, SOD and glutathione transferase (GST) enzymes activities in liver by 27, 130 and 168 percent, respectively. Plasma nitrate concentration and Haemoglobin levels remained the same in all the treatments. Feeding of high CLA ghee resulted in lower (P < 0.01) plasma cholesterol & triglyceride level (52.17 and 30.27%), and higher high density lipoproteins (33.26%) than feeding of soybean oil (control group) and thus manifested in decreased (P < 0.05) atherogenic index (from 0.472 to 0.244). Lesser cholesterol and triglyceride levels were observed in the liver and aorta of high CLA fed rats than in those of the other groups. Histopathological studies of liver showed normal hepatic cords with portal triad in the high CLA ghee fed rats whereas fatty degeneration of hepatocytes containing fat vacuoles was observed in the liver of the other groups.

**Conclusion:**

This paper is the first report of the antioxidant and antiatherogenic properties of the high CLA enriched ghee suggesting that high CLA ghee can be used as a potential food for decreasing the risk of cardiovascular diseases, particularly in India, where, ghee is widely used for culinary and medicinal purposes.

## Background

Hypercholesterolemia, obesity and oxidation stress are the major risk factors that, alone or in combination, accelerate the development of cardiovascular diseases and the progression of atherosclerosis [1]. Due to insoluble nature of cholesterol in blood, it is transported to and from the cells by carriers known as lipoproteins- Low-density lipoprotein (LDL) and High-density lipoprotein (HDL). People with high triglyceride level along with high LDL level and a low HDL level are at health risk [2]. Antioxidants are the molecules that inhibit the oxidation of other molecules and these include glutathione, vitamin C, vitamin A, and vitamin E as well as enzymes such as catalase, superoxide dismutase and various peroxidases [3]. Water-soluble antioxidants react with oxidants (free radicals) in the cell cytosol and the blood plasma, while lipid-soluble antioxidants protect the cell membranes from lipid peroxidation.

Conjugated linoleic acid (CLA) is an important food component that consists of a mixture of positional and geometric isomers of linoleic acid (*cis −*9, *cis −*12, C_18:2_) with two conjugated double bonds at various carbon positions in the fatty acid chain [4]. CLA is formed as an intermediate during the biohydrogenation of linoleic acid by linoleic acid isomerase from the rumen bacteria *Butyrivibrio fibrisolvens*[5] or from the endogenous conversion of *t*-11, C_18:1_ (vaccenic Acid), another intermediate of linoleic or linolenic acid biohydrogenation, by Δ^9^-desaturase in the tissues [6]. Milk fat is the richest natural dietary source of CLA which contains an average 4.5 mg CLA/g of fat [7]. CLA is considered as an important biologically-active compound of food due to its proven anticarcinogenic, antiallergic and anti-inflammatory properties [8,9].

The relationship between food and health has long been known to exist, and today the fundamental concept of food is changing from one involving the maintenance of life to one maintaining and promoting better health and quality of life by preventing chronic diseases. As mentioned earlier, hypercholesterolemia, obesity and oxidative stress are the major risk factors for developing atherosclerosis and cardiovascular diseases in humans. Although numerous synthetic drugs are available for decreasing cholesterol levels [10], still there are a lot of side effects associated with these drugs. This anomaly has renewed research interests in developing new strategies to use natural components for maintaining cholesterol levels with least side effects. Hence, taking these factors into consideration, the aim of the present work was to study the effect of CLA enriched ghee (clarified butter) on the antioxidant enzyme activities of blood and liver of CLA fed Wistar rats. In addition to this, antiatherogenic activity in aorta and liver of Wistar rats was also studied for maintaining health.

## Results

### Fatty acids composition in ghee

Fatty acid composition in all the three treatment groups has been summarized in Table 1. Short chain fatty acids (C_4:0_ to C_12:0_) and medium chain fatty acids (C_14:0_ to C_16:1_) contents were comparable in all the three groups. The stearic acid (C_18:0_) level was lower (P < 0.01) in high CLA ghee (137.05 mg/g fat) than in other groups. Significant differences exist in the level of trans vaccenic acid in all the groups. The level of *cis-9, trans-11* fatty acid level in high CLA ghee was 169 and 69 percent higher than that in other fats. The level of *trans-10, cis-12,* the other isomer of CLA in corresponding groups was 0.68, 1.61 and 2.78 mg/g fat, and difference among the groups was significant (P < 0.05). The Omega-3-fatty acids content was higher (P < 0.05) in low CLA ghee and high CLA ghee than in soybean oil whereas Omega 6 fatty acids level was similar in all the three groups and values. The ratio of Omega 6: Omega 3 fatty acids was lowest in high CLA ghee and highest in soybean oil.

**Table 1:** Average fatty acid composition (mg/g fat) of dietary fats

**Fatty acid**	**Soybean oil**	**Low CLA ghee**	**High CLA ghee**
C4:0	31.84 ± 0.32	31.52 ± 0.16	31.82 ± 0.66
C6:0	16.60 ± 0.10	17.38 ± 0.78	16.83 ± 0.29
C8:0	8.72 ± 0.19	9.41 ± 0.48	8.75 ± 0.55
C10:0	16.77 ± 0.46	18.06 ± 0.28	17.73 ± 0.33
C12:0	23.43 ± 0.55	24.44 ± 0.45	23.47 ± 0.67
C14:0	105.21 ± 1.66	108.55 ± 0.86	106.15 ± 0.41
C14:1	5.42 ± 0.11	5.45 ± 0.07	5.81 ± 0.30
C16:0	265.03 ± 1.68	269.39 ± 0.53	275.3 ± 0.61
C16:1	13.40 ± 0.33	13.86 ± 0.31	14.89 ± 0.74
C18:0*	143.11^a^ ± 2.65	139.27^b^ ± 1.06	137.05^c^ ± 1.02
C18:1 9-t	1.73 ± 0.18	1.92 ± 0.03	1.96 ± 0.13
C18:1 9-c	241.13 ± 2.27	240.87 ± 2.14	244.65 ± 3.13
C18:1 11-c (VA)*	9.65^a^ ± 0.48	13.12 ^b^ ± 0.06	16.63 ^c^ ± 0.09
C18:2 9-c,12-c	11.87 ± 0.44	11.47 ± 0.23	11.44 ± 0.17
C20:0	11.54 ± 0.47	10.27 ± 0.29	10.05 ± 0.32
C18:3 6-9-12-c	0.04 ± 0.04	0.00 ± 0.00	0.00 ± 0.00
C18:3 9-12-15-c	3.42 ± 0.06	9.44 ± 0.12	9.12 ± 0.04
Total C18:3	3.46 ± 0.09	9.44 ± 0.12	9.12 ± 0.04
C18:2 9-c,11-t**	6.23^a^ ± 0.07	10.57^b^ ± 0.16	16.76^c^ ± 0.22
C18:2 10-t,12-c*	0.68 ^a^ ± 0.10	1.61 ^b^ ± 0.16	2.78^c^ ± 0.16
Total CLA**	6.92^a^ ± 0.04	12.18^b^ ± 0.02	19.54^c^ ± 0.07
Total Omega 3 FA*	4.30 ^a^ ± 0.10	10.42^b^ ± 0.23	10.54^b^ ± 0.08
Total Omega 6 FA	13.09 ± 0.51	12.73 ± 0.33	12.52 ± 0.21
Omega 6 : Omega3*	3.05 ^a^ ± 0.14	1.22^b^ ± 0.03	1.18^b^ ± 0.09

Values are Mean ± SE for n = 5.

Figures with different superscript across a row differ significantly (*P < 0.05; ** P < 0.01).

### Effect of feeding experimental diets on antioxidant enzyme (AOE) activities in blood

Maximum CAT activity was observed in high CLA ghee group, followed by low CLA ghee group (Table 2). Catalase activity was increased by 48, 33 and 12% in high CLA, low CLA ghee and soybean oil fed group, respectively, compared to day zero (21st day) and variation among the groups was significant (P < 0.01). Similarly, in case of SOD activity, maximum SOD activity was observed in high CLA ghee fed group followed by low CLA fed group and lowest in soybean oil fed group. SOD activity increased to 41.70, 22.74 and 8% in high CLA, low CLA ghee and soybean oil fed group, respectively, compared to day zero and variation among the groups was significant (P < 0.01). Plasma nitric oxide level increased to significant levels in different dietary groups (day 35) as compared to control (day 0). Hemoglobin content was not much affected by the different treatments.

**Table 2:** Effect of feeding soybean oil, low CLA and high CLA ghee diet on selected parameters

**Before treatment (on Day 0)**		**Experimental trials (on day 35)**		
	**Feed**	**Soybean oil**	**Low CLA ghee**	**High CLA ghee**
Average feed intake (gram/day/animal)	6.67^b^ ± 0.08	11.02^a^ ± 0.13	11.22^a^ ± 0.19	10.92^a^ ± 0.12
Aveage body weight of rats (g)	33.33^b^ ± 1.47	97.43^a^ ± 2.13	99.57^a^ ± 2.10	98.00^a^ ± 2.47
**Blood (RBC lysate)**				
CAT (U/mg Hb)	57.67^d^ ± 1.07	64.5^c^ ± 1.63	76.83^b^ ± 1.57	85.50^a^ ± 1.72
SOD (U/mg Hb)	3045.50^d^ ± 55.30	3301.67^c^ ± 106.70	3738.33^b^ ± 57.01	4317.50^a^ ± 54.32
Plasma nitric oxide (mmol/ml)	3.85^b^ ± 0.15	8.33^a^ ± 0.24	8.28^a^ ± 0.29	7.92^a^ ± 0.12
Hemoglobin (mg/dL)	11.12^a^ ± 0.40	11.04^a^ ± 0.50	12.34^a^ ± 0.42	12.45^a^ ± 0.38
**Liver Enzymes**				
CAT (U/mg protein)	156.33^d^ ± 2.83	187.87^c^ ± 8.27	215.36^b^ ± 7.49	238.01^a^ ± 6.85
SOD (U/mg protein)	2.85^d^ ± 0.09	4.07 ^c^ ± 0.38	7.98^b^ ± 0.36	9.38^a^ ± 0.38
GST (U/mg protein)	0.180^d^ ± 0.02	0.279^c^ ± 0.01	0.531^b^ ± 0.01	0.749^a^ ± 0.02
Protein (mg/g tissue)	62.00^b^ ± 1.08	69.71^a^ ± 1.12	69.29^a^ ± 1.45	70.14^a^ ± 0.98
Lipid peroxides (TBARS) (nmol/g tissue)	12.24^d^ ± 0.48	26.23^a^ ± 0.44	20.54^b^ ± 0.50	18.71^c^ ± 0.53

Values are Mean ± SE for n = 8.

Values in rows with different superscript are significantly different (P < 0.05).

### Effect of feeding experimental diets on antioxidative enzymes in liver

At the age of 55^th^ day, all the animals were sacrificed and CAT, SOD and GST activities were analyzed in liver. Liver CAT activity was the highest in high CLA ghee fed group followed by low CLA fed group and lowest in soybean oil fed group (Table 2). CAT activity increased to 52.24, 37.82 and 20% in high CLA, low CLA ghee and soybean oil fed group, respectively, from that of its day zero and variation among the groups was significant (P < 0.05). Similarly, highest liver SOD activity was determined in high CLA ghee fed group followed by low CLA fed group and lowest in soybean oil fed group. SOD activity was increased by 227, 179 and 42% in high CLA, low CLA ghee and soybean oil group, respectively, from zero day and variation among the groups was significant (P < 0.05).

The detoxifying, phase II GST enzyme was the highest in high CLA ghee fed group followed by low CLA fed group and lowest in soybean oil fed group (Table 2). Significant variations were observed among the different dietary groups (P < 0.01). GST activity increased significantly in high CLA, low CLA ghee and soybean oil fed group, respectively, compared to day zero. The extent of endogenous lipid peroxidation level of TBARS (Thiobarbituric acid reactive substances) was the highest in soybean oil fed group followed by low CLA fed group and lowest in high CLA ghee fed group (Table 2). TBARS levels significantly changed at day 35 (P < 0.01) as compared to controls (day zero) in different dietary groups.

### Effect of experimental diets on antiatherogenic potency in rats

The average fortnight feed intake increased with an increase in body weight in all the dietary treatment groups (Table 3). The results revealed that there was no significant variation in the feed intake as well as body weight gain among the different treatments (Table 3). The total cholesterol level was higher (P < 0.05) in soybean oil fed group as compared to low CLA and high CLA ghee diet. This result indicated that high and low CLA ghee diets had 52.17 and 26.06% reduction in total cholesterol than the soybean oil diet. The highest level of HDL was recorded in high CLA ghee diet fed group and lowest HDL in soybean oil diet group (Table 3).

**Table 3:** Effect of feeding soybean oil, low CLA ghee and high CLA ghee diets on the lipid profile in Rats

**Parameters studied**	**Soybean oil diet**	**Low CLA ghee diet**	**High CLA ghee diet**
**Average feed intake (gram/day/animal)**	23.03 ± 2.41	22.75 ± 2.53	22.78 ± 2.53
**Body weight (gram)**			
0th day^NS^	131.13 ± 4.70	125.00 ± 2.85	128.57 ± 3.45
120 days^NS^	263.33 ± 4.81	265.00 ± 6.67	262.12 ± 4.21
**Plasma total cholesterol (mg/dL)**			
0th day^NS^	58.09 ± 1.24	57.68 ± 2.11	58.82 ± 1.99
120 days^NS^	81.33^a^ ± 2.91	74.40^b^ ± 1.68	69.93^c^ ± 1.44
**Plasma HDL-cholesterol (mg/dL)**			
0th day^NS^	34.72 ± 0.78	35.65 ± 1.15	34.60 ± 1.10
120 days^NS^	35.51^a^ ± 0.91	41.84^b^ ± 0.54	47.32^c^ ± 0.96
**Plasma triglycerides (mg/dL)**			
0th day^NS^	40.87 ± 0.77	41.41 ± 0.88	40.44 ± 0.75
120 days^NS^	73.23^a^ ± 1.08	65.01^b^ ± 1.16	56.21^c^ ± 0.42
**Plasma LDL-cholesterol (mg/dL)**			
0th day^NS^	14.26 ± 1.72	14.67 ± 2.38	13.84 ± 2.10
120 days^NS^	26.79^a^ ± 3.53	19.57^b^ ± 1.86	14.38^c^ ± 1.62
**Atherogenic Index**			
0th day^NS^	0.410 ± 0.06	0.430 ± 0.08	0.472 ± 0.07
120 days^NS^	0.971^a^ ± 0.12	0.570^b^ ± 0.05	0.244^c^ ± 0.04

Values are Mean ± SE for n = 8.

Values in rows/columns with different superscript differ significantly (P < 0.01),*(P < 0.05).

Maximum increase in the triglyceride level was observed in soybean oil fed group as compared to low CLA followed by high CLA ghee fed group. This result indicated that high CLA and Low CLA ghee diets fed groups had 30.27 and 12.64% reduction in triglyceride than the soybean oil fed group. The LDL level was increased in all the three groups and highest level was observed in soybean oil fed group and lowest in high CLA fed group. Soybean oil fed group animals had 86.30 and 36.89% higher LDL compared to the high CLA and low CLA groups. After the feeding period, maximum AI was found (P < 0.01) in soybean oil fed group as compared to low and high CLA ghee fed groups. The high CLA ghee diet reduced the AI index from 0.472 to 0.244 whereas soybean oil increased AI from 0.410 to 0.971 in rats. Additional files 1, 2, 3, 4 and 5 show the trend of increase/decrease of a particular parameter at 30 days interval time period (See Additional files 1, 2, 3, 4 and 5).

### Effect of feeding experimental diets on tissue lipids

**Figure 1:**
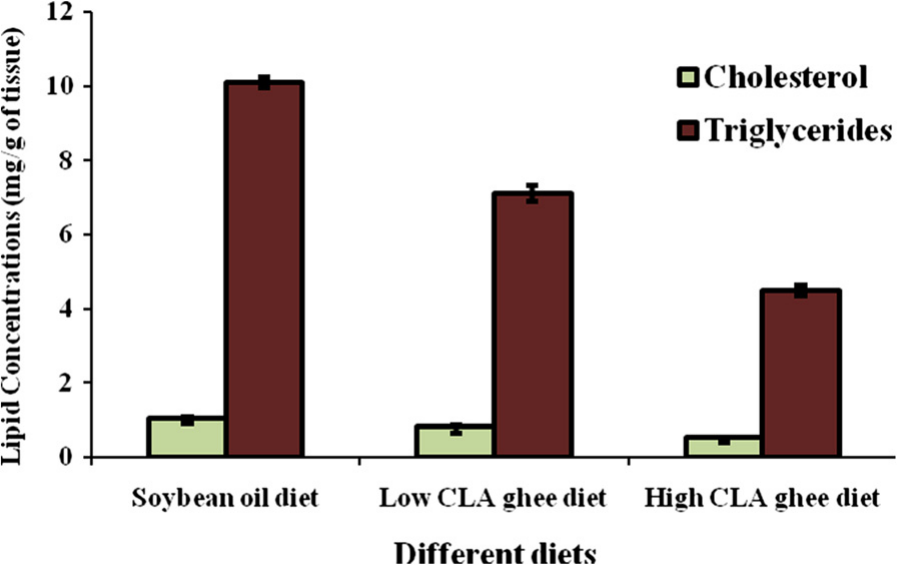
**Effect of different diets on the lipid profile of liver tissue of weanling female Wistar rats after 120 days of experiment.** Values are Mean ± SE for n = 8.

**Figure 2:**
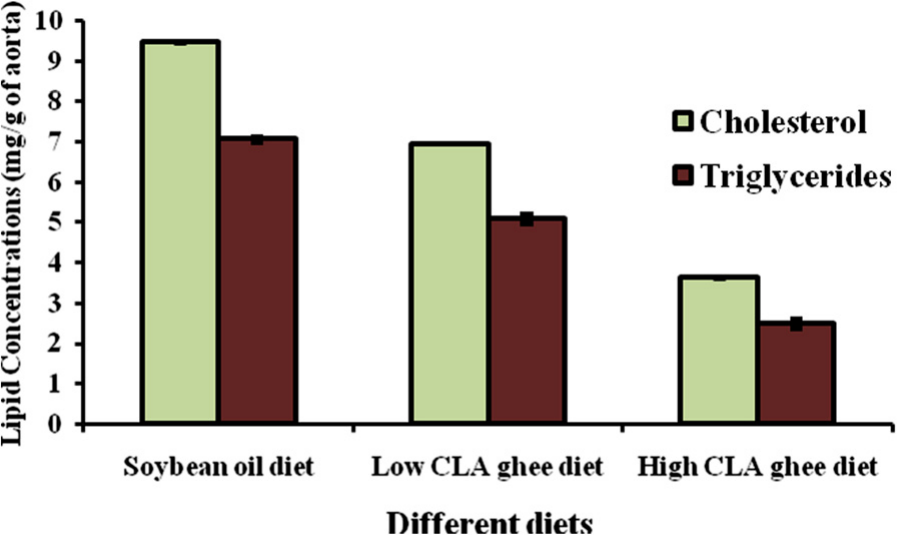
**Effect of different diets on the lipid profile of aorta of weanling female Wistar rats after 120 days of experiment.** Values are Mean ± SE for n = 8.

Maximum cholesterol and triglyceride level was observed in soybean oil fed group whereas lowest amount was detected in high CLA ghee fed group in both liver and aorta (Figures 1 and 2). Significant variations were observed among the groups (P < 0.01).

### Histopathology of liver

**Figure 3:**
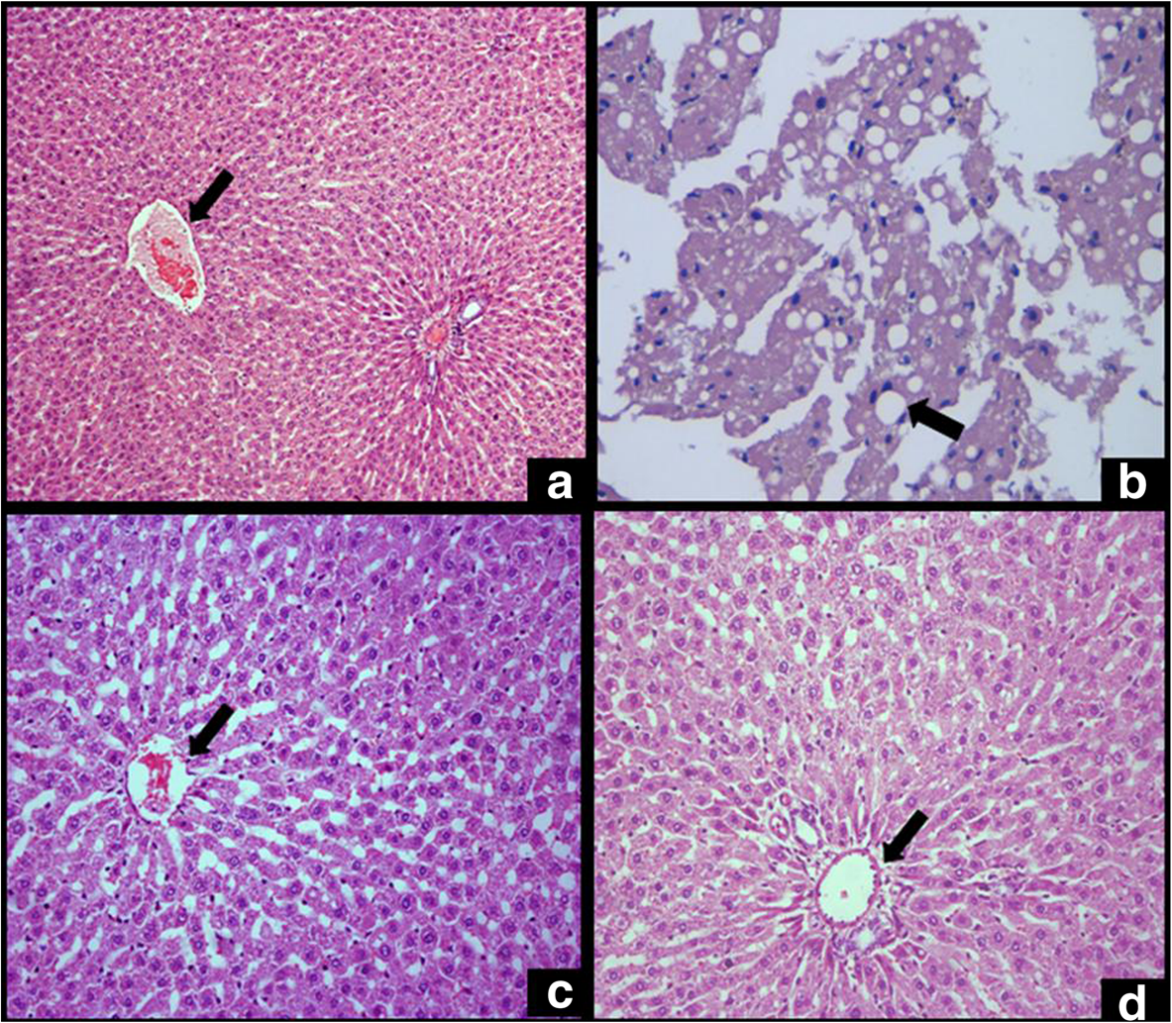
**Histopathological studies of liver of rats. (a)** Normal rat’s (rats not fed with experimental diet) liver (100×), **(b)** Soybean oil fed rat’s liver (400×), **(c)** Low CLA ghee fed rat’s liver (200×), **(d)** High CLA ghee fed rat’s liver (200×).

Histopathology of liver was done to study the deposition of fat in hepatocytes and sinusoids. The normal architecture of liver showed central vein surrounded by hepatocytes along with portal triad containing hepatic artery, bile duct and portal vein (Figure 3a). In soybean oil diet fed group, severe fatty degeneration of hepatocytes containing fat vacuoles was observed in the histopathological study. Due to degeneration of hepatocytes, the nuclei were seen in the periphery of vacuole (Figure 3b). Liver of low CLA ghee diet fed rats showed mild degeneration of hepatocytes surrounding the central vein (Figure 3c) whereas in high CLA ghee fed rats showed normal hepatic cords with portal triad (Figure 3d).

## Discussion

The objective of this study was to assess the effect of CLA containing food product (ghee) on antioxidative enzyme system and to study the antiatherogenic property in the blood and liver of rats. The results indicated that feeding of low and high CLA ghee diets during pubescent period (from 21^st^ day to 55^th^ day) increased 20 & 33% CAT and 13 & 28% SOD activity, respectively, in blood as compared to the group fed on soybean oil diet. Similarly, an increased liver CAT and SOD activities were observed in high CLA group as compared to soybean oil fed group. Hence, higher CAT & SOD activities in group fed with high CLA ghee diet may offer higher protection to the cells against oxidants. Similar results were reported by Santos Zago et al., [11], wherein they showed that administration of CLA to the diet of rats increased the catalase and lipid peroxidases, which finally lead to an improvement in the antioxidative process. Higher CAT and SOD activities in response to CLA diet given to rats have also been reported by Ip et al. [12] and Bhatia [13]. Thus, in our work, ghee with high CLA has enhanced the activity of CAT and SOD enzymes by scavenging the free radicals which ultimately improves the antioxidative enzyme systems. In pubescent development of rats, the level of nitric oxide was observed to be the same in all the treatment groups. Not much significant variations were observed in the treatment groups; which clearly shows that nitric oxide is independent of CLA containing diet. Haemoglobin, too, is not much affected by the CLA content in the diet. Not much significant effect on haemoglobin was observed by Steck et al. [14] in case of rats administered with CLA content.

In the present study, GST activity was the highest in high CLA ghee followed by low CLA ghee fed groups rather than in soybean oil fed group. Higher GST activity in response to high CLA are believed to modify the antioxidant system in rats [15,16].

Feeding of low and high CLA ghee from weaning to sexual maturity of rats lowered 27 and 40% lipid peroxidation than the soybean oil fed rats in present study. Lipid peroxidation is considered as the main molecular mechanisms involved in the oxidative damage to cell structures and in the toxicity process that lead to cell death [17]. Oils have been reported to induce a significant increase in the antioxidant enzyme activities and a decrease in the conjugated dienes (CD) and thiobarbituric acid-reactive substances levels in the liver [18]. Thus high CLA fed diet lowers the lipid peroxidation process and inturn protects the cells from its harmful effects. Recent study by Varady et al. [19] has proved that oxidized fat increases the nuclear concentration of NF-κB and Nrf2 and transcript levels of oxidative stress-responsive genes coding for aldo–keto reductase 1B8, vanin-1, glutathione peroxidase 1, and superoxide dismutase-1. Hence, CLA plays a definite role in taking care of oxidative stress in rats and removing the free radicals from the biological system.

Several animal studies have established that CLA has profound effects on lipoprotein metabolism and it prevents diet-influenced atherosclerosis. Hence the experiment was conducted to study the antiatherogeneic potency of CLA in adult female (9 weeks old) rats by feeding CLA for 120 days. Not much significant variations in the average body weight of rats fed with different dietary treatments were observed. This proves that CLA has anti-obesity property, which may be attributed to the modulation in lipid metabolism leading to the reduction of lipid uptake by adipocytes and manifested as reduction in body fat gain [2]. Yu et al. [20] reported that two weeks feeding of *trans*-10, *cis*-12 CLA reduced abdominal adipose tissue weight than linoleic acid fed to rats. These effects were attributable to suppressed fatty acid synthesis and enhanced beta-oxidation of fatty acid in the liver on *trans*-10, *cis-*12 CLA diet.

In our study, low and high CLA feeding reduced total cholesterol by 52.17 and 26.06%, triglycerides by 23.00 and 11.00%, however, HDL increased by 17.82 and 33.26% than soybean oil feeding in rats. The supplementation of diet containing CLA has shown a reduction in the levels of serum total cholesterol and total triglyceride and increased high-density lipoprotein cholesterol level as compared to control diet groups without CLA [21,22]. In another study by Bauchart et al. [23] butters varying in *trans* 18:1 and *cis*-9,*trans-*11 Conjugated Linoleic Acid has modified Plasma Lipoproteins in the Hypercholesterolemic Rabbit. Present results clearly indicated that CLA feeding, irrespective of its level in diet, narrowed the atherogenic index (0.472 to 0.244) in rats than those fed on soybean oil (0.410 to 0.971) by decreasing LDL and increasing HDL.

Our results also indicated that the presence of CLA in the diet of rats inhibited deposition of cholesterol and triglycerides by two and three folds, respectively, in liver as well as in aorta and the response of CLA was depending upon its level in the diet. Fatty acid composition and presence of polar lipids in the diets have significant effects on lipid partitioning, gene expression, and potentially the development of liver pathology in mice [24]. In a recent study by Tan et al. [25], they have proved that CLA addition significantly increases the Carnitine palmitoyltransferase I enzyme levels (that catalyzes an important regulatory step in lipid metabolism) which ultimately results in the reduction of lipid contents both in liver and muscle tissues.

As discussed earlier, dietary CLA tends to modulate lipid metabolism in rats. In the present study, soybean oil feeding resulted in more fat deposition in hepatocytes compared to the CLA feeding (Plate 4.1B). Recent work by Kostogrys and Pisulewski [26] have also discussed about the alterations in the histopathology of liver tissues and concluded that CLA in high-fructose diet, decreases serum LDL + VLDL and trigylcerides and plasma malondialdehyde concentrations as well as liver weight and liver cholesterol, thus opposing the effects of high-fructose diet and showing a potential antiatherogenic effect.

## Conclusion

The study shows that high CLA enriched ghee increases the antioxidant enzyme activities such as CAT, SOD and GST activities; which concludes its antioxidant nature. Feeding of high CLA enriched ghee also lead to a decrease in cholesterol, triglyceride and high density lipoproteins and inturn, decreased the low density lipoproteins, which finally lead to the reduction in atherogenic index, which proves its antiatherogenic property. The present study indicates that high CLA enriched ghee (clarified butter) has antioxidant and antiatherogenic activities suggesting that ghee can be as an important food component for decreasing the risk of cardiovascular diseases.

## Methods

Protocols were conducted in accordance with standard ethical guidelines, it was approved by the IAEC (Institutional Animal Ethics Committee).

### Preparation of ghee

Low CLA (12.18 mg/g fat) and high CLA (19.54 mg/g fat) ghee was prepared by creamery method from milk of buffaloes fed on groundnut oil cake and mustard cake plus 2% mustard oil based concentrates, respectively [8]. Briefly, milk was heated to 40°C and the cream was separated by centrifugation in a cream separator. The cream was heated to 115°C in a stainless steel jacket ghee kettle fitted with an agitator, steam control valve, pressure and temperature gauges and a movable, hollow, stainless steel tube centrally bored for emptying out the contents. Heating was discontinued as soon the color of the ghee residue turned to golden yellow.

### Experimental animals, housing and feeding

Twenty four female rats of Wistar strain (21 days old) with body weight of 25–42 g were obtained from small animal house of National Dairy Research Institute, Karnal, Haryana, India and housed in metal cages in an air conditioned room at 24 ± 1°C with 12:12 h light/dark cycle. Rats were randomly divided into 3 groups (n = 8) namely Soybean oil, low CLA ghee and High CLA ghee diet groups (Table 4). Experimental diets were fed for a period of 35 days for studying antioxidative enzymes. For studying the antiatherogenic activity, animals were randomly divided into 3 groups (n = 8) and fed with the same diet as soybean oil, low CLA and high CLA diet (Table 4) as described above for 16 weeks. The water and diet was given to animals on *adlibitum* basis. Animals were weighed at fortnight intervals.

**Table 4:** Composition of diets (g/kg of diet)

**Component**	**Soybean oil diet**	**Low CLA ghee diet**	**High CLA ghee diet**
Bengal gram	540.0	540.0	540.0
Wheat	130.0	130.0	130.0
Groundnut cake	60.0	60.0	60.0
Soybean oil	200.0	-	-
Low CLA ghee	-	200.0	-
High CLA ghee	-	-	200.0
Skimmed milk powder	44.4	44.4	44.4
Mineral mixture	21.6	21.6	21.6
Vitamin mixture	2.0	2.0	2.0
Choline chloride	2.0	2.0	2.0

### Effect of feeding on antioxidative enzymes (AOE) system

#### Collection and processing of blood

Blood was collected before starting the experimental diet feeding. i.e. on zero day and on fortnightly basis till the completion of experiment. Haemoglobin from blood was estimated using hemoglobin reagent by Drabkin’s Cynanomethemoglobin method [27]. RBC lysate was prepared and antioxidative enzymes *viz,* catalase [28], superoxide dismutase [29] were analyzed. Nitric oxide estimation from plasma was done as per Nims et al. [30]. One international CAT Unit (U) is equivalent to 1 μmol of H_2_O_2_ consumed/minute/mg of protein at 25°C. One international SOD unit is equivalent to the amount of enzymes that inhibit the reaction upto 50%.

#### Collection of liver

All the rats were sacrificed after the experiment and entire liver was excised. Liver homogenate was prepared as described by Cohen et al. [31]. For catalase assay, liver homogenate was further processed as described by Cohen et al. [31] and the catalase activity and Superoxide dismutase were assayed. Glutathione-S-Transferase (GST) was determined by the method of Habig et al. [32]. One international GST unit is expressed as mmoles of cDNB conjugated/minute/mg protein. For detection of lipid peroxides (malondialdehyde) in liver homogenate method of Uchiyama and Mihara [33] was followed. Protein was estimated according to the method of Bradford [34].

### Effect of feeding on antiatherogenic potency in rats

For studying the antiatherogenic potency, twenty four female albino rats of Wistar strain (8 to 9 weeks old, 125–150 g) were housed in metal cages in an air conditional room at 24 ± 1°C in small animal house of National Dairy Research Institute, Karnal and housed. Animals were randomly divided into 3 groups of eight animals each and fed with the same diet as soybean oil, low CLA and high CLA diet (Table 4) as described above for 16 weeks. The water and diet was given to animals on *adlibitum* basis. Animals were weighed at fortnight intervals.

#### Analysis of lipids in blood

Blood was collected before starting the experimental diet feeding. i.e. on zero day and on fortnightly basis till the completion of experiment. Immediately after collection, hemoglobin content in blood was estimated [27] and rest of the blood was centrifuged at 3500 rpm for 10 minutes at 4°C in a refrigerated centrifuge (Sigma Laborzentrifuge, Model 1 K15). Plasma was separated and stored at −20°C till further analysis. Total Cholesterol, HDL and triglycerides were analyzed using Autopak kits (Bayer Diagnostic India Ltd., Gujarat, India). LDL and Atherogenic Index (AI) were calculated using friedewalds’s equation.

$$\begin{array}{l} \mathrm{LDL}\;\mathrm{Cholesterol}\left(\mathrm{mg}/\mathrm{dl}\right)=\mathrm{Total}\;\mathrm{cholesterol}\\ \;-\frac{\mathrm{Triacylglycerol}}{5}\\ \;-\mathrm{HDL}\;\mathrm{Cholesterol} \end{array}$$

$$\mathrm{Atherogenic}\;\mathrm{Index}\left(\mathrm{AI}\right)=\frac{\mathrm{LDL}\;\mathrm{cholesterol}}{\mathrm{HDL}\;\mathrm{cholesterol}}$$

#### Analysis of lipids in liver tissue

All the rats were sacrificed after the study period and entire liver and aorta were excised, washed with isotonic buffer and stored at −70°C. Liver homogenate was prepared and total cholesterol and triglycerol in the extracted fat samples of liver and aorta were estimated using Autopak kits (Bayer Diagnostic India Ltd., Gujarat, India).

### Histopathology of tissues

The washed tissue was dehydrated in the ascending grades of isopropanol and finally cleared in xylene. The tissue was then embedded in molten paraffin wax. Sections were cut at 5 mm thickness and stained with Hematoxylin and Eosin [35].

### Statistical analyses

Results are expressed as Mean ± SE unless otherwise stated. Statistical comparisons were made by Univariate General Linear Model, T-test and Mann–Whitney test using IBM SPSS software, version 20.

## Abbreviations

AI: Atherogenic Index; AOE: Antioxidative Enzymes; CAT: Catalase; CD: Conjugated dienes; CDNB: 1-Chloro,2,4-DinitroBenzene; CLA: Conjugated linoleic acid; GST: Glutathione transferase; HDL: High density lipids; LDL: Low density lipids; NOS: Nitric oxide synthase; SOD: Superoxide dismutase; TBARS: Thiobarbituric acid reactive substances; VLDL: Very low density lipids.

## Competing interests

The authors report no competing interests.

## Authors’ contributions

KC and AKT designed the research. KC performed experiments. HKK and KC analyzed data and wrote the paper. HKK, KC, AKT, CS and PR support and review the study. All authors have read and approved the final manuscript.

## Supplementary Material

Additional file 1 Plasma total cholesterol (mg/dL) levels in rats fed on Soybean oil/Low CLA ghee/high CLA ghee diet. Click here for file

Additional file 2 Plasma HDL-cholesterol (mg/dL) levels in rats fed on Soybean oil/ Low CLA ghee/high CLA ghee diet. Click here for file

Additional file 3 Plasma triglycerides (mg/dL) levels in rats fed on Soybean oil/Low CLA ghee/high CLA ghee diet. Click here for file

Additional file 4 Plasma LDL (mg/dL) levels in rats fed on Soybean oil/Low CLA ghee/high CLA ghee diet. Click here for file

Additional file 5 Atherogenic Index in rats fed on Soybean oil/Low CLA ghee/high CLA ghee diet. Click here for file
