# Supplementary material for: High conjugated linoleic acid enriched ghee (clarified butter) increases the antioxidant and antiatherogenic potency in female Wistar rats
Source: Lipids Health Dis. 2013 Aug 7;12:121. doi: 10.1186/1476-511X-12-121 (PMC3766171; doi:10.1186/1476-511X-12-121)
Supplement: Additional file 1 — Plasma total cholesterol (mg/dL) levels in rats fed on Soybean oil/Low CLA ghee/high CLA ghee diet. [file 1476-511X-12-121-S1.doc]

**Additional file1:** Plasma total cholesterol (mg/dL) levels in rats fed on Soybean oil/Low CLA ghee/high CLA ghee diet

| **Days** | **Groups** | | |
| --- | --- | --- | --- |
|  | **Soybean oil** | **Low CLA ghee** | **High CLA ghee** |
| 0^NS^ | 58.09 ± 1.24 | 57.68 ±2.11 | 58.82 ± 1.99 |
| 30* | 76.29 ^a^ ± 1.85 | 72.73^b^ ± 1.83 | 65.92^c^ ± 1.32 |
| 60* | 78.45 ^a^ ± 2.65 | 73.00^b^ ± 1.32 | 67.61^c^ ± 1.08 |
| 90 | 81.26^a^ ± 1.09 | 75.11^b^ ± 1.22 | 68.84^c^ ± 1.14 |
| 120 | 81.33^a^ ± 2.91 | 74.40 ^b^ ± 1.68 | 69.93^c^ ± 1.44 |

Values are Mean±SE for n=8

Values in rows with different superscript differ significantly *(P<0.05) (P<0.01)
