# Supplementary material for: High conjugated linoleic acid enriched ghee (clarified butter) increases the antioxidant and antiatherogenic potency in female Wistar rats
Source: Lipids Health Dis. 2013 Aug 7;12:121. doi: 10.1186/1476-511X-12-121 (PMC3766171; doi:10.1186/1476-511X-12-121)
Supplement: Additional file 2 — Plasma HDL-cholesterol (mg/dL) levels in rats fed on Soybean oil/ Low CLA ghee/high CLA ghee diet. [file 1476-511X-12-121-S2.doc]

**Additional file2:** Plasma HDL-cholesterol (mg/dL) levels in rats fed on Soybean oil/ Low CLA ghee/high CLA ghee diet

| Days | **Groups** | | |
| --- | --- | --- | --- |
|  | **Soybean oil** | **Low CLA ghee** | **High CLA ghee** |
| 0^NS^ | 34.72 ± 0.78 | 35.65 ±1.15 | 34.60± 1.10 |
| 30 | 35.79^a^ ± 0.79 | 39.22^b^ ± 0.98 | 42.60^b^ ±1.02 |
| 60 | 35.23^a^ ± 1.11 | 40.41^b^ ±11.10 | 44.96^c^ ± 0.94 |
| 90 | 34.85^a^ ± 0.75 | 40.73^b^ ± 0.82 | 46.82^c^ ± 0.87 |
| 120 | 35.51^a^ ± 0.91 | 41.84^b^ ± 0.54 | 47.32^c^ ± 0.96 |

Values (mg/dL) are Mean±SE for n=8

Values in rows with different superscript differ significantly (P<0.01)
