# Supplementary material for: High conjugated linoleic acid enriched ghee (clarified butter) increases the antioxidant and antiatherogenic potency in female Wistar rats
Source: Lipids Health Dis. 2013 Aug 7;12:121. doi: 10.1186/1476-511X-12-121 (PMC3766171; doi:10.1186/1476-511X-12-121)
Supplement: Additional file 3 — Plasma triglycerides (mg/dL) levels in rats fed on Soybean oil/Low CLA ghee/high CLA ghee diet. [file 1476-511X-12-121-S3.doc]

**Additional file3:** Plasma triglycerides (mg/dL) levels in rats fed on Soybean oil/Low CLA ghee/high CLA ghee diet

| **Days** | **Groups** | | |
| --- | --- | --- | --- |
|  | **Soybean oil** | **Low CLA ghee** | **High CLA ghee** |
| 0^NS^ | 40.87 ± 0.77 | 41.41± 0.88 | 40.44 ± 0.75 |
| 30 | 65.52^a^ ± 1.53 | 60.89 ^b^± 1.03 | 56.01^c^ ± 1.01 |
| 60 | 71.52^a^ ± 0.92 | 63.54^b^± 0.96 | 58.50^c^ ± 0.81 |
| 90 | 71.55^a^ ± 0.98 | 64.54^b^± 1.04 | 54.02^c^ ± 1.14 |
| 120 | 73.23^a^ ± 1.08 | 65.01^b^± 1.16 | 56.21^c^ ± 0.42 |

Values (mg/dL) are Mean±SE for n=8

Values in rows with different superscript differ significantly (P<0.01)
