# Supplementary material for: High conjugated linoleic acid enriched ghee (clarified butter) increases the antioxidant and antiatherogenic potency in female Wistar rats
Source: Lipids Health Dis. 2013 Aug 7;12:121. doi: 10.1186/1476-511X-12-121 (PMC3766171; doi:10.1186/1476-511X-12-121)
Supplement: Additional file 4 — Plasma LDL (mg/dL) levels in rats fed on Soybean oil/Low CLA ghee/high CLA ghee diet. [file 1476-511X-12-121-S4.doc]

**Additional file4:Plasma LDL (mg/dL) levels in rats fed on Soybean oil/Low CLA ghee/high CLA ghee diet**

| **Days** | **Groups** | | |
| --- | --- | --- | --- |
|  | **Soybean oil** | **Low CLA ghee** | **High CLA ghee** |
| 0 | 14.26 ± 1.72 | 14.67 ± 2.38 | 13.84 ± 2.10 |
| 30* | 24.66^a^ ± 1.95 | 20.49^b^ ± 1.23 | 17.01^b^ ± 1.85 |
| 60 | 25.92^a^ ±3.19 | 19.87^b^ ± 1.30 | 15.09^c^ ± 1.35 |
| 90 | 26.33^a^ ±1.73 | 18.84^b^ ± 1.46 | 14.33^c^ ± 1.14 |
| 120 | 26.79^a^ ± 3.53 | 19.57^b^ ± 1.86 | 14.38^c^ ± 1.62 |

Values (mg/dL) are Mean±SE for n=8

Values in rows with different superscript differ significantly (P<0.01),*(P<0.05)
