# Supplementary material for: High conjugated linoleic acid enriched ghee (clarified butter) increases the antioxidant and antiatherogenic potency in female Wistar rats
Source: Lipids Health Dis. 2013 Aug 7;12:121. doi: 10.1186/1476-511X-12-121 (PMC3766171; doi:10.1186/1476-511X-12-121)
Supplement: Additional file 5 — Atherogenic Index in rats fed on Soybean oil/Low CLA ghee/high CLA ghee diet. [file 1476-511X-12-121-S5.doc]

**Additional file5:** Atherogenic Index in rats fed on Soybean oil/Low CLA ghee/high CLA ghee diet

| **Days** | **Groups** | | |
| --- | --- | --- | --- |
|  | **Soybean oil** | **Low CLA ghee** | **High CLA ghee** |
| 0 ^NS^ | 0.410 ± 0.06 | 0.430 ± 0.08 | 0.472 ± 0.07 |
| 30 | 0.737^a^ ± 0.06 | 0.592^b^± 0.03 | 0.418^c^ ± 0.05 |
| 60 | 0.841^a^ ± 0.11 | 0.560 ^b^± 0.04 | 0.313^c^ ± 0.03 |
| 90 | 0.997^a^ ± 0.05 | 0.532^b^± 0.04 | 0.288^c^ ± 0.04 |
| 120 | 0.971^a^ ± 0.12 | 0.570^b^ ± 0.05 | 0.244^c^± 0.04 |

Values are Mean ± SE for n=8

Values in rows with different superscript differ significantly (P<0.01)
